# TSGA10 as a Model of a Thermal Metabolic Regulator: Implications for Cancer Biology

**DOI:** 10.3390/cancers17111756

**Published:** 2025-05-23

**Authors:** Ali Amini, Farzad Taghizadeh-Hesary, John Bracht, Babak Behnam

**Affiliations:** 1 Center for Data Science, American University, 4400 Massachusetts Avenue NW, Washington, DC 20016, USA; 2 Ear, Nose, Throat, and Head and Neck Research Center and Department, The Five Senses Health Institute, School of Medicine, Iran University of Medical Sciences, Tehran 14496-14535, Iran; 3 Biology Department, American University, 4400 Massachusetts Avenue NW, Washington, DC 20016, USA; 4 Avicenna Biotech Research, Germantown, MD 20854, USA

**Keywords:** TSGA10, mitochondrial coupling, Complex III (CytC1), carcinogenesis, HIF-1α, Warburg effect

## Abstract

TSGA10, a multifunctional protein critical for mitochondrial coupling and metabolic regulation, plays a paradoxical role in cancer progression and carcinogenesis. Here, we outline a potential mechanism by which TSGA10 mediates metabolism in oncogenesis and thermal modulation. Initially identified in spermatogenesis, TSGA10 interacts with mitochondrial Complex III: it directly binds cytochrome c1 (CytC1). In our model, TSGA10 optimizes electron transport to minimize reactive oxygen species (ROS) and heat production while enhancing Adenosine Triphosphate (ATP) synthesis. In cancer, TSGA10’s expression is context-dependent: Its downregulation in tumors like glioblastoma might disrupt mitochondrial coupling, promoting electron leakage, ROS accumulation, and genomic instability. This dysfunction would be predicted to contribute to a glycolytic shift, facilitating tumor survival under hypoxia. Conversely, TSGA10 overexpression in certain cancers suppresses HIF-1α, inhibiting glycolysis and metastasis. TSGA10 and HIF-1α engage in mutual counter-regulation—TSGA10 represses HIF-1α to sustain oxidative phosphorylation (OXPHOS), while HIF-1α suppression of TSGA10 under hypoxia or thermal stress amplifies glycolytic dependency. This interplay is pivotal in tumors adapting to microenvironmental stressors, such as cold-induced mitochondrial uncoupling, which mimics brown adipose tissue thermogenesis to reduce ROS and sustain proliferation. Tissue-specific TSGA10 expression further modulates cancer susceptibility: high levels in the testes and brain may protect against thermal and oxidative damage, whereas low expression in the liver permits HIF-1α-driven metabolic plasticity. Altogether, our model suggests that TSGA10 plays a central role in mitochondrial fidelity. We suggest that its crosstalk with oncogenic pathways position it as a metabolic rheostat, whose dysregulation fosters tumorigenesis through ROS-mediated mutagenesis, metabolic reprogramming, and microenvironmental remodeling. Targeting the hypothesized TSGA10-mediated mitochondrial coupling may offer therapeutic potential to disrupt cancer’s adaptive energetics and restore metabolic homeostasis.

## Simple Summary

We outline a potential mechanism by which Testis-Specific Gene 10 (TSGA10) helps mitochondria produce energy efficiently while reducing harmful byproducts like reactive oxygen species (ROS) and heat. By interacting with cytochrome c1 (CytC1) in mitochondrial Complex III, TSGA10 may stabilize electron transfer, minimizing leakage to oxygen and reducing ROS and heat production. Our model predicts that in cancer, TSGA10 acts as a double-edged sword: Low levels allow ROS buildup and force cancer cells to rely on less efficient energy methods (like glycolysis), promoting tumor growth. Conversely, high TSGA10 levels can suppress tumors by maintaining mitochondrial health and blocking cancer-friendly pathways. TSGA10 and Hypoxia-inducible factor 1-alpha (HIF-1α—a protein that helps cancer cells thrive in low oxygen) regulate each other, creating a balance that shapes tumor behavior. TSGA10’s varying levels in tissues—high in the brain and testes (protecting against damage) and low in the liver (enabling cancer growth)—make it a promising target for therapies aimed at disrupting cancer’s energy strategies.

## 1. Introduction

TSGA10, a gene encoding a 698-amino acid protein, was first identified in the testis and is involved in spermatogenesis, cytoskeletal organization, and metabolic regulation [1,2]. It undergoes alternative splicing, producing multiple transcript variants, and is expressed in tissues such as the brain, retina, and germ cell tumors [3,4]. Processed into N-terminal (27-kD) and C-terminal (55-kD) components, TSGA10 localizes to sperm and is expressed in other tissues, including the liver, brain, and kidney [3,5]. It interacts with Hypoxia-inducible factor 1-alpha (HIF-1α), modulating its nuclear localization and transcriptional activity, and inhibits cancer cell growth, motility, and invasion by downregulating HIF-1α target genes like Vascular Endothelial Growth Factor A (*VEGFA*), Matrix Metalloproteinase-2 (*MMP2*), and Matrix Metalloproteinase-9 (*MMP9*) [6,7]. TSGA10 also interacts with vimentin, a cytoskeletal protein involved in oxygen sensing [8].

TSGA10 is overexpressed in cancers such as melanoma, colon cancer, hepatocellular carcinoma, ovarian cancer, prostate cancer, and leukemia [9,10]. Paradoxically, its downregulation in some contexts is also linked to cancer progression, highlighting its dual role as both a tumor suppressor and potential oncogene [2]. Recent studies suggest that TSGA10 regulates mitochondrial organization and perinuclear translocation, potentially regulating metabolic activity [2,11], positioning it as a key modulator of cellular energy metabolism and stress responses.

Mitochondria, beyond their role in ATP production, are sophisticated regulators of stress responses, calcium signaling, and reactive oxygen species (ROS) production and signaling [12]. They act as molecular thermostats, modulating energy dissipation as heat through controlled coupling and uncoupling of chemiosmotic gradients, which are critical for tissue-specific metabolic demands and systemic temperature homeostasis.

Here, we propose that TSGA10, highly expressed in metabolically active cells, may optimize mitochondrial coupling efficiency by interacting with CytC1, enhancing electron transport through Complex III, promoting ATP synthesis, and minimizing electron leak, heat production, and ROS generation [2]. This mechanism may enable cells to adapt to fluctuating oxygen levels by switching between oxidative phosphorylation and glycolysis.

TSGA10’s high expression in neurons suggests a critical role in protecting against thermal stress. Neurons are highly vulnerable to heat due to their complex structure, temperature-sensitive ion channels, and reliance on ATP-dependent processes, including synaptic function and membrane stability [13,14,15]. Thermal stress disrupts oxidative phosphorylation, increases ROS production, and compromises mitochondrial efficiency, leading to neuronal dysfunction and degeneration [16,17]. Neurons’ limited regenerative capacity makes thermal-induced damage potentially permanent [18,19]. TSGA10 may enhance neuronal thermoregulation by optimizing mitochondrial coupling efficiency and reducing ROS levels, which is particularly vital in the brain, given its high metabolic demands and susceptibility to thermal stress. TSGA10’s role at the intersection of mitochondrial regulation and environmental adaptation highlights its potential as a therapeutic target for neurodegenerative diseases and cancer.

## 2. Materials and Methods

The yeast strain AH109 was transformed with pGBKT7-TSGA10 and a rat testis library. Positive clones were identified using selective media, and TSGA10-prey gene expression was confirmed via PCR. CYTC1 emerged as a strong interactor, validated through sequencing and galactosidase activity assays. Additionally, yeast mating was performed with PGAD424-transformed Y187 yeast, and confirmed cDNA inserts were sequenced for accuracy.

To visualize the interaction, NIH3T3 cells were cultured and transfected with GFP-tagged TSGA10 using Lipofectamine (Invitrogen, Carlsbad, CA, USA) 3000. After 24–48 h, cells were stained with MitoTracker Red CMXRos Thermo Fisher Scientific, Cat. No. M7512, Waltham, MA, USA) to label mitochondria, and then fixed with 4% paraformaldehyde.

Using confocal microscopy, colocalization of GFP-TSGA10 (green) and mitochondria (red) appeared as yellow regions in merged images. Image quantification was performed using Zeiss LSM 510 i-UV laser scanning confocal microscope (Carl Zeiss AG, Oberkochen, Germany).

Mitochondrial fractions were isolated from NIH3T3 cells via differential centrifugation. The mitochondrial pellet was resuspended in RIPA buffer with protease inhibitors. Lysates were pre-cleared with protein A/G beads, and then incubated overnight with a mouse monoclonal anti-CytC1 antibody. Captured protein complexes were washed, eluted, and then analyzed via SDS-PAGE and Western blot. The membrane was probed with a rabbit anti-TSGA10 antibody to detect TSGA10 within the CytC1 immunoprecipitate and an anti-GFP antibody to identify GFP-TSGA10 fusion proteins and their proteolytic fragments (27 kDa N-terminus and 55 kDa C-terminus). Chemiluminescence was used for visualization.

To ensure specificity, a negative control using an irrelevant mouse IgG antibody was included in Co-IP, while untransfected or empty vector-transfected NIH3T3 cells served as controls for fluorescent imaging.

## 3. Mitochondrial Role in Heat Production and Uncoupling

### 3.1. Mitochondrial (De-)Coupling

In coupled mitochondria, the electron transport chain generates a proton gradient that drives ATP synthesis with high efficiency, while uncoupled mitochondria dissipate this gradient energy as heat through regulated proton leak mechanisms [20]. Decoupling of mitochondria is generally thought of as a way to slow down ATP production when ROS accumulate as a toxic byproduct [21]. However, decoupling is also a mechanism by which tissues can increase their operating temperature. This complex balance gives cells a control point for adjusting ATP, ROS, and temperature to meet the metabolic requirements of each tissue. Given that blood flow is also a thermal distribution system regulated by the central nervous system, the organism can use temperature both as input (to mediate metabolism) and as output (using mitochondrial uncoupling to alter local thermogenesis). In this way, an organism can raise or lower the thermal set-point of each tissue, embodying a shifting medley of metabolic states over time.

### 3.2. Mitochondrial Coupling Across Healthy Tissues

Mitochondrial metabolism exhibits a spectrum of coupling efficiency across tissues within the organism. At one end, spermatocytes in the testis prioritize tightly coupled oxidative phosphorylation (OXPHOS) to support their high energy needs during meiosis, while keeping temperatures low. Efficient ATP production via coupled mitochondria ensures genomic stability, but even this efficient process generates some ROS (which inescapably increases with OXPHOS activity), posing risks to DNA integrity. To mitigate this, spermatocytes employ robust antioxidant systems, crucial in the cooler testicular environment (2–8 °C below core body temperature), where lower thermal energy may reduce enzymatic repair efficiency [22].

### 3.3. Mitochondrial Decoupling in Brown Adipose Tissue

In stark contrast, brown adipose tissue (BAT) dissipates proton gradients to produce heat instead of ATP via the expression of uncoupling protein 1 (UCP1). The inverse relationship between coupling efficiency and thermal output highlights a metabolic trade-off: ATP synthesis versus heat. Cancer cells, facing microenvironmental stress, exploit metabolic plasticity. Under cold stress, they may adopt BAT-like uncoupling to reduce ROS and generate heat, sustaining proliferation. Concurrently, cold impairs OXPHOS enzymes like Complex III, promoting ROS accumulation and a glycolytic shift akin to the Warburg effect [23]. This dual adaptation—uncoupling for survival and glycolysis for ATP—reflects metabolic reprogramming under thermal duress [24]. Thus, tissue-specific mitochondrial strategies, from spermatocyte coupling to BAT uncoupling and cancer’s hybrid approach, illustrate how metabolic pathways change across and within tissues to meet physiological and environmental demands.

### 3.4. Mitochondrial Uncoupling and Cancer

With emerging evidence linking metabolic reprogramming, heat production, and uncoupling to carcinogenesis, mitochondria play a complex role in cancer. Cancer cells often exhibit mitochondrial uncoupling, a process where proton leakage across the inner mitochondrial membrane reduces ATP synthesis efficiency, diverting energy into heat production (thermogenesis). This uncoupling, mediated by proteins like UCP1/UCP2, may support tumor survival by mitigating oxidative stress (ROS) or adapting to hypoxic microenvironments. Excessive ROS from dysfunctional electron transport chains can damage DNA and drive oncogenic mutations, while uncoupling-induced heat might alter the tumor microenvironment, promoting angiogenesis or metastasis. The Warburg effect (aerobic glycolysis) and mitochondrial uncoupling together reflect a metabolic shift that prioritizes rapid energy and biomass production over efficiency, enabling cancer progression. Thus, mitochondrial thermogenesis and uncoupling are not merely metabolic quirks but potential contributors to tumorigenesis and therapeutic targets.

## 4. TSGA10 and Metabolic Activity

**Figure 1:**
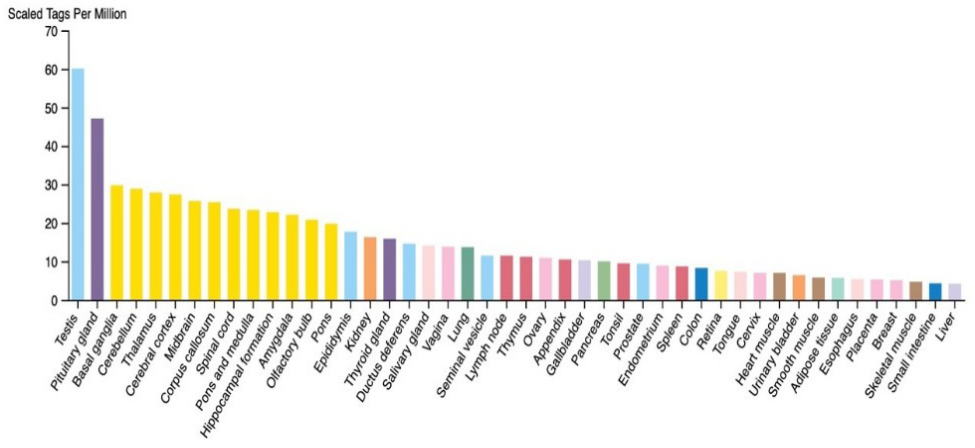
RNA expression level of TSGA10 in different human tissues (courtesy of the open-source The Human Atlas Protein [26]). Colors indicate tissue types, with male reproductive being light blue and neuronal being yellow.

TSGA10 exhibits context-dependent roles in metabolic regulation across postmitotic cells and cancer, with its expression levels aligning with distinct cellular energy demands. *TSGA10* expression across human tissues appears to show elevated expression in thermosensitive and energy-demanding tissues (e.g., testis and brain) and reduced levels in thermally resilient tissues (e.g., liver), reflecting a potential role in mitochondrial coupling and metabolic adaptation (Figure 1). Consistent with this observation, postmitotic cells like spermatocytes exhibit high TSGA10 expression, which also correlates with improved sperm quality [25]. This aligns with the high energy demands of spermatozoa, which rely on mitochondrial OXPHOS for motility. Similarly, in other postmitotic cells (e.g., neurons and nephrons), TSGA10’s suppression of HIF-1α [6] may promote oxidative metabolism over glycolysis, ensuring efficient ATP production in these non-proliferative, long-lived cells.

In contrast, cancer cells exhibit a metabolic inconsistency. TSGA10 overexpression in breast cancer suppresses HIF-1α-mediated glycolysis and metastatic activity [27], effectively countering the Warburg effect (aerobic glycolysis). This suggests that high TSGA10 levels in tumors are associated with reduced glycolytic flux and attenuated malignancy. However, in hepatocellular carcinoma, TSGA10’s link to drug resistance via lncRNAs [28] implies a more complex metabolic interplay, potentially involving stress-induced adaptations.

Overall, consistency across some cell types is observed. In postmitotic cells, the upregulated TSGA10 may support oxidative metabolism (e.g., in ciliary-riched spermatozoa and inferred neurons/nephrons), consistent with their reliance on OXPHOS. Also, in cancer cells, we predict that higher TSGA10 expression suppresses glycolysis (via HIF-1α inhibition), aligning with reduced tumor aggressiveness. Conversely, low TSGA10 may permit glycolytic dominance, fueling proliferation.

### 4.1. Transcriptional and Post-Transcriptional Regulation of TSGA10

The dynamic expression of TSGA10 across developmental stages and tissues underscores its context-dependent roles, which are tightly regulated by transcriptional and post-transcriptional mechanisms. Transcriptional regulation of TSGA10 involves nuclear transcription factor Y (NF-Y), which binds to the CCAAT box in its promoter to activate expression, as demonstrated in zebrafish and conserved in mammals [4,29]. This aligns with findings that NF-Y often regulates cell cycle-related genes, supporting TSGA10’s potential role in mitosis and DNA repair via its conserved SMC domain. Epigenetic modifications further fine-tune TSGA10 expression. For instance, DNA hypomethylation at the TSGA10 promoter has been linked to its overexpression in cancers like transitional cell carcinoma [30], while hypermethylation in other contexts may suppress its expression, contributing to tissue-specific regulation. Histone modifiers, such as HDACs, may also modulate TSGA10 chromatin accessibility, though this warrants further investigation [31].

Post-transcriptional control mechanisms, particularly miRNA-mediated regulation, play pivotal roles in adapting TSGA10 levels to environmental stimuli. Hypoxia-induced miR-10b-3p directly targets TSGA10, promoting angiogenesis and metastasis in esophageal squamous cell carcinoma [32]. Similarly, exosomal miR-23a in nasopharyngeal carcinoma downregulates TSGA10 to enhance vascularization [25]. These findings highlight a conserved feedback loop where hypoxia-driven HIF-1α not only induces angiogenic miRNAs but also suppresses TSGA10, which itself inhibits HIF-1α nuclear localization [6]. This reciprocal regulation allows TSGA10 to act as a hypoxia-responsive brake on angiogenesis, balancing pro- and anti-tumorigenic signals.

Environmental stressors like hypoxia and hormonal signals further diversify TSGA10’s regulatory landscape. In the testis, TSGA10’s constitutive expression is likely maintained by androgen-responsive elements [25], whereas in cancers, oxidative stress and metabolic changes may alter its epigenetic or miRNA-mediated regulation. Such plasticity enables TSGA10 to adapt its roles—from supporting spermatogenesis in normal testis to suppressing angiogenesis in hypoxic tumors or promoting developmental patterning in embryos. Future studies should explore tissue-specific enhancers, lncRNAs, and additional post-translational modifications that may refine TSGA10’s functional versatility in response to diverse stimuli.

### 4.2. TSGA10 and Mitochondria

**Figure 2:**
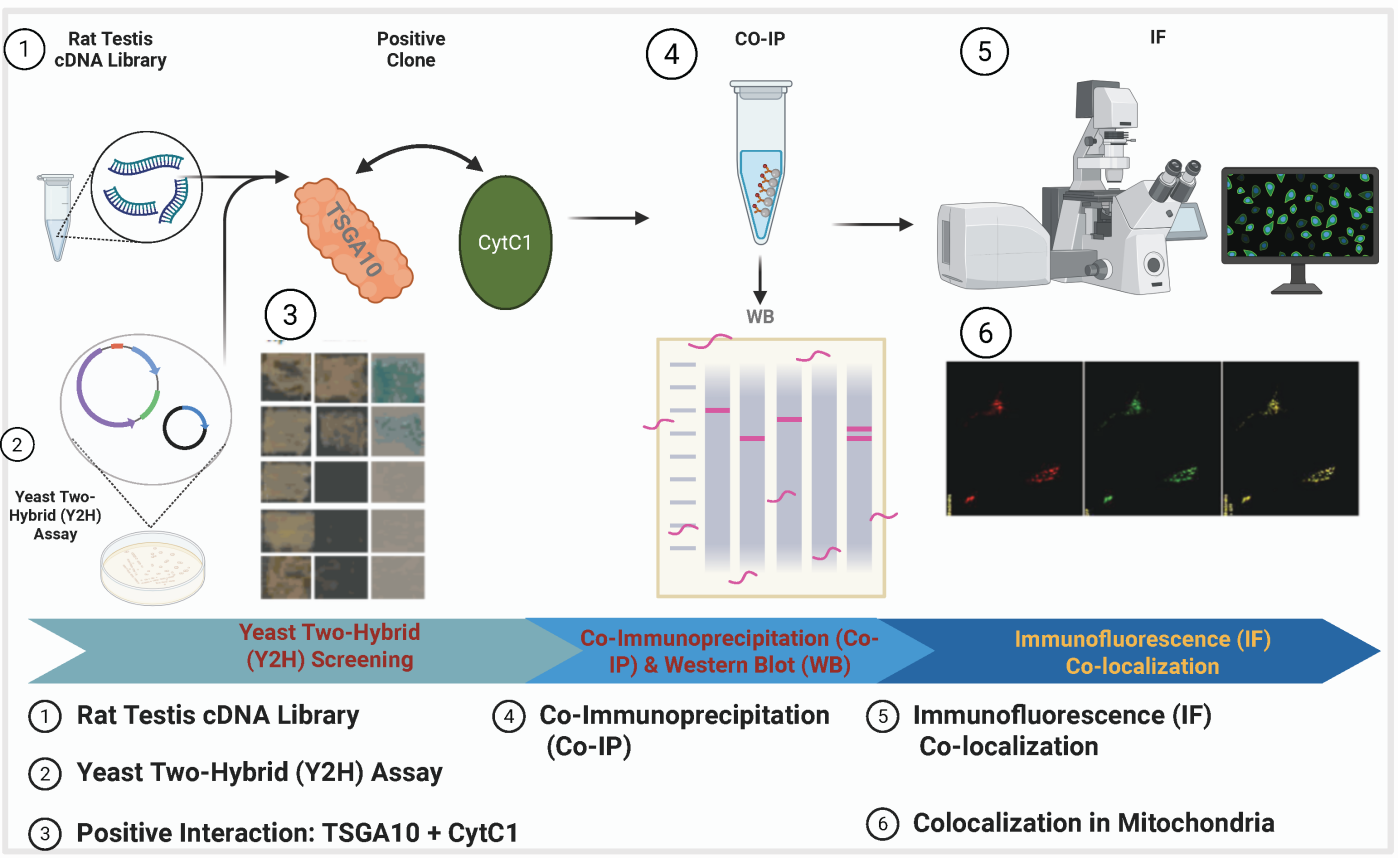
TSGA10 protein association with mitochondria in NIH3T3 cells. This schematic outlines the experimental workflow to demonstrate TSGA10’s association with mitochondria, where TSGA10 interacts with CytC1. The process includes a yeast two-hybrid (Y2H) assay; TSGA10 is used as the bait protein to screen a testis cDNA library, leading to the identification of CytC1 as an interacting partner. Then, the interaction is validated in mammalian cells through co-immunoprecipitation (CO-IP) followed by Western blot (WB); CytC1 antibody is used for immunoprecipitation, and the presence of TSGA10 in the complex is confirmed using TSGA10 and GFP antibodies during WB. Finally, a immunofluorescence (IF) colocalization study isperformed to visualize the subcellular localization of TSGA10 and CytC1 in mitochondria. Together, these experiments confirm the physical interaction and mitochondrial co-localization of TSGA10 and CytC1.

TSGA10 is strongly associated with mitochondrial function, particularly in the context of spermatogenesis and male fertility. TSGA10 was shown to be essential for the proper arrangement of the mitochondrial sheath during spermatid differentiation [11]. TSGA10 has been shown to colocalize with mitochondria [11,33], shown in Figure 2, to bind to the mitochondrial protein NSUN2 [34].

TSGA10 is critical for sperm development and mitochondrial organization. Disruption of TSGA10 function led to defects in mitochondrial organization, resulting in male infertility. Similarly, the loss-of-function mutations in TSGA10 cause acephalic spermatozoa syndrome in humans, characterized by abnormal sperm head formation and mitochondrial sheath disorganization, further underscoring its role in mitochondrial integrity during spermatogenesis [35]. Additionally, a connection between TSGA10 and autophagy has been explored [36], suggesting that TSGA10 may regulate mitochondrial quality control through autophagy pathways, which is vital for maintaining mitochondrial function during sperm maturation. Collectively, these studies highlight TSGA10 as a key factor in mitochondrial organization and function, particularly in the context of male fertility, where its proper expression is crucial for the structural and functional integrity of sperm mitochondria.

**Figure 3:**
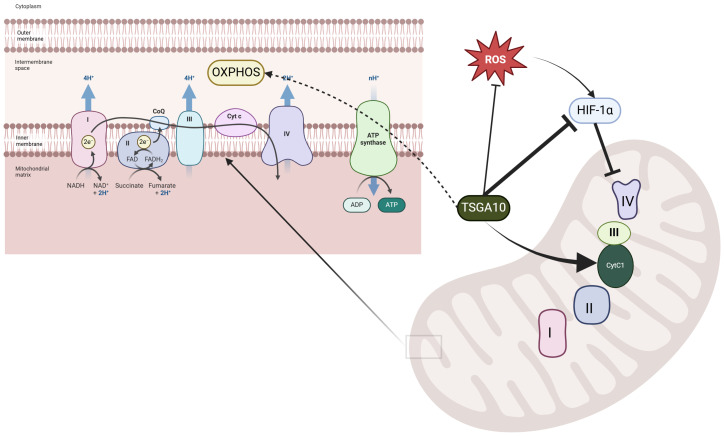
Proposed role of TSGA10 interaction with cytochrome c1 in mitochondria. The left panel illustrates the electron transport chain (ETC) in the mitochondrial inner membrane, where Complexes I–IV facilitate electron transfer and proton pumping to generate ATP through oxidative phosphorylation.

TSGA10 is not merely associated with mitochondria; it directly interacts with cytochrome c1 (CytC1), a subunit of Complex III in the mitochondrial electron transport chain (ETC) [33]. The interaction between TSGA10 and CytC1 was discovered by one of us (B. Behnam) by a yeast two-hybrid (Y2H) screening assay and confirmed by both colocalization and co-immunoprecipitation [33]. This functional interaction suggests that TSGA10 may play a crucial role in mitochondrial energy metabolism or regulation. Figure 2 illustrates the comprehensive experimental validation of this interaction, demonstrating TSGA10’s mitochondrial association through multiple complementary techniques. The combined evidence from yeast two-hybrid screening, co-immunoprecipitation studies, and immunofluorescence assays collectively establishes TSGA10 as a bona fide mitochondrial-interacting protein with specific affinity for CytC1. CytC1 directly transfers an electron from cytochrome b1 to cytochrome c1 [37], and we therefore propose that TSGA10 is a previously unknown metabolic regulator, leveraging its interaction with cytochrome c1 (CytC1) within Complex III of the electron transport chain to stabilize OXPHOS and modulate ROS production (Figure 3).

The right panel highlights the regulatory role of TSGA10 in mitochondrial function, where it interacts with cytochrome c1 (Cyt C1) of Complex III, potentially modulating electron transport efficiency. TSGA10 is also implicated in the suppression of HIF-1α, a key transcription factor involved in hypoxia adaptation, which can downregulate Complex IV activity. Additionally, TSGA10 may influence ROS production, linking its function to oxidative stress responses. The black arrows indicate proposed direct interactions, while dashed lines represent indirect or putative pathways.

### 4.3. TSGA10 and Its Potential Role in Mitochondrial Coupling

#### 4.3.1. Mitochondrial Complex III and the Electron Transport Chain

As a subunit of Complex III in the mitochondrial ETC, CytC1 plays a critical role in transferring electrons from ubiquinol to cytochrome c. Complex III is an electrically driven proton pump in which subunit b1 mediates electron flow from ubiquinol along with proton uptake and release; CytC1 is involved in the terminal transfer of the electron to cytochrome c [38] (Figure 3).

The interaction between TSGA10 and CytC1 suggests that TSGA10 may have a role in modulating the function of Complex III in electron transfer to cytochrome c. Therefore, a plausible hypothesis for TSGA10’s role in mitochondrial coupling can be proposed. We hypothesize that TSGA10 directly influences mitochondrial coupling by adjusting electron transfer efficiency, modulating the choice between the transfer of electrons to cytochrome c versus oxygen and ROS production. In essence, we propose that TSGA10 is an extra insulator ensuring the electron moves to cytochrome c instead of leaking to oxygen prematurely (i.e., before Complex IV, where it is supposed to combine with oxygen in a coordinated response, minimizing ROS production and generating water) (Figure 3). One could hypothesize that TSGA10 acts positively (leading to stronger coupling and lower ROS production) or negatively (leading to weaker coupling and increased ROS). Given that TSGA10 is expressed in energy-demanding cells, we propose that its role is to strengthen the coupling of electron flow within OXPHOS (i.e., promoting electron flow into cytochrome c and minimizing ROS production), thereby improving mitochondrial efficiency in ATP generation [2,39]. This regulatory role could be crucial for maintaining cellular energy homeostasis, particularly in tissues with high metabolic demands, such as the testis, where TSGA10 is predominantly expressed. However, the mechanistic details of how TSGA10 might do this remain to be investigated.

#### 4.3.2. A Potential Thermal Role for TSGA10

There is an important thermal component to TSGA10’s potential coupling function, which arises by minimizing the inefficiencies associated with electron leakage and ROS production (and as discussed earlier in Section 3, “Mitochondrial Role in Heat Production and Uncoupling”). When electron flux is high, excess electrons escape the ETC in a process known as electron leakage, contributing to ROS generation [21]. This premature donation of an electron to oxygen generates a superoxide anion, a potent ROS, but it also short-circuits the electron transport chain [40]. As discussed in Section 3 earlier, brown adipose tissue uses UCP1 to decouple the proton gradient, allowing protons to diffuse back across the inner mitochondrial membrane. Not only does this produce heat by non-shivering thermogenesis, but it also slows down an overactive ETC, thereby reducing toxic ROS production generated by electron leakage [41]. This protective effect is thermogenic: short-circuiting the ETC releases energy that would ordinarily go to making ATP; this energy produces heat instead [20].

A direct implication of the preceding discussion is that by binding Complex III and reducing electron leakage, TSGA10 could minimize both ROS and the need for uncoupling of the mitochondrial proton gradient. In turn, this would reduce the production of uncoupling-associated heat. TSGA10 thus would act as a cooling and coupling agent for the mitochondria. This mechanism could be particularly advantageous in scenarios where thermal regulation is essential, such as in protecting cells from thermal stress or in adapting to environments with limited energy resources.

#### 4.3.3. A Potential Complex III Assembly Role for TSGA10

We have outlined a mechanism of direct regulation, but another possibility is that TSGA10 plays a role in the assembly of Complex III itself. Rip1, a Rieske Fe/S subunit of Complex III, is essential for electron transfer [42], and its proper assembly relies on the multi-protein mitochondrial contact site and cristae organizing system (MICOS) [43]. In particular, Mar26/Fmp10 coordinates Complex III assembly by linking Rieske Fe/S protein-containing intermediates to crista junctions for Rip1 maturation [43]. We hypothesize that TSGA10 functions similarly, helping to construct OXPHOS-competent Complex III.

#### 4.3.4. Disruption of Mitochondrial Coupling in Health and Disease

Dysregulation of mitochondrial coupling is implicated in diseases such as cancer, neurodegeneration, and metabolic syndromes. If TSGA10 dysfunction disrupts coupling efficiency, it could contribute to these pathologies. Conversely, therapeutic targeting of the TSGA10-CytC1 axis might restore apoptosis and autophagy/mitophagy in cancer, and energy balance in mitochondrial disorders. For instance, enhancing TSGA10’s activity could improve ATP synthesis in energy-deficient conditions, while inhibiting it might mitigate pathologies driven by excessive coupling.

Moreover, TSGA10’s proposed role in optimizing mitochondrial electron transfer and coupling through Complex III may hold significant implications for cancer biology. By stabilizing electron flow to cytochrome c and minimizing leakage to oxygen, TSGA10 could suppress ROS generation and reduce heat production—a critical balance disrupted in carcinogenesis. Cancer cells often exhibit mitochondrial dysfunction, including elevated ROS, which drives genomic instability and oncogenic signaling. If TSGA10 expression is compromised, inefficient electron transfer at Complex III could exacerbate ROS leakage, promoting DNA damage and mutations that fuel tumorigenesis. Furthermore, TSGA10’s potential role in Complex III assembly, akin to the mitochondrial contact site and cristae organizing system (MICOS)-dependent Rip1 maturation, suggests that its dysfunction might impair OXPHOS integrity, forcing cells to rely on glycolytic metabolism (the Warburg effect), a hallmark of cancer. Conversely, tumoral cells, especially the tumors in energy-demanding tissues (e.g., testicular cancers) might exploit TSGA10’s coupling efficiency to sustain ATP production while evading ROS-induced apoptosis. The thermal component of TSGA10’s function—reducing heat from electron leakage—could also influence the tumor microenvironment, where excess heat may promote angiogenesis or immune evasion. Thus, TSGA10 emerges as a potential metabolic gatekeeper whose dysregulation could link mitochondrial coupling defects, ROS-driven mutagenesis, and metabolic reprogramming in cancer progression, offering novel therapeutic targets to disrupt tumor energetics.

### 4.4. TSGA10 and Oxygen Sensing

Previous work has shown that TSGA10 also interacts with HIF-1α [6]. This positions TSGA10 as an oxysensor or member of the oxygen sensor complex protein, including HIF-1α, Complex III (bc1 complex), intermediate filament network, and TSGA10 itself, to modulate an appropriate response to stress and hypoxia. The mitochondrial Complex III regulates hypoxic activation of HIF-1α via enabling cells to sense hypoxia. Under normoxic conditions, HIF-1α is rapidly degraded by prolyl hydroxylases (PHDs), which require oxygen as a substrate. However, during hypoxia, Complex III of the electron transport chain generates ROS as a byproduct of incomplete oxygen reduction. These ROS inhibit PHD activity, stabilizing HIF-1α and allowing it to translocate to the nucleus. There, HIF-1α dimerizes with HIF-1β, activating the transcription of genes involved in angiogenesis, metabolism, and survival, thereby enabling cellular adaptation to hypoxia [44]. Given our hypothesis that TSGA10 directly affects electron transfer either to cytochrome c or oxygen (thereby generating the ROS involved in HIF-1α activation), it is positioned to be a direct player in the process of oxygen sensing by HIF-1α and Complex III.

### 4.5. HIF-1α in Thermoregulation

As described above, the interaction between HIF-1α, TSGA10, CytC1, and mitochondrial metabolism may play a role in dynamically adjusting energy production and thermoregulation in response to cellular and environmental stressors. While HIF-1’s role in metabolism is well established, emerging evidence suggests that hypoxia-inducible factors can modulate thermogenic pathways. For instance, HIF-2α has been shown to function as a thermostat in beige adipocytes by regulating PKA activity and mitochondrial abundance in response to cold and re-warming [45]. These findings raise the intriguing possibility that HIF-1α may both respond to temperature and regulate mitochondrial heat production, acting as a molecular thermostat in conjunction with the TSGA10 feedback loop outlined above.

## 5. TSGA10 and HIF-1α Mutual Counter Repression in Thermoregulation

### 5.1. HIF-1α-Dependent Regulation of ROS Generation and Thermogenic Mechanisms in Hypoxic Conditions

HIF-1α has been demonstrated to play a crucial role in ROS generation, particularly under hypoxic conditions [46,47,48]. The landmark study by Chandel et al. [46] established that mitochondrial ROS are not only elevated during hypoxia but are essential for HIF-1α stabilization and activity, creating a feedback loop in which HIF-1α and ROS regulate each other. Multiple mechanisms underlying this relationship have been identified: HIF-1α reprograms mitochondrial metabolism under hypoxia, leading to electron leakage and enhanced ROS production at Complex III of the electron transport chain [47]; it transcriptionally induces pro-oxidant enzymes such as the NADPH oxidases (NOX family), which directly generate ROS [48]; and while HIF-1α downregulates mitochondrial respiration through mediators like pyruvate dehydrogenase kinase 1 (PDK1), this metabolic shift paradoxically causes transient mitochondrial dysfunction and ROS bursts during cellular adaptation. Guzy and Schumacker [47] specifically addressed this paradox of increased ROS generation during hypoxia, while Semenza’s review [48] highlighted the significance of these HIF-1α-mediated mechanisms in cancer progression and potential therapeutic interventions.

Although HIF-1α does not directly increase heat generation as a primary function, it indirectly contributes to thermogenesis through multiple pathways under specific physiological and pathological conditions [49]. HIF-1α upregulates glycolytic enzymes and glucose transporters (e.g., GLUT1), favoring anaerobic metabolism, which, despite being less efficient for ATP production, generates more heat per mole of ATP than oxidative phosphorylation—particularly evident in tumors and inflammatory states [50]. In brown adipose tissue, HIF-1α may influence thermogenesis by modulating the expression of uncoupling protein 1 (UCP1), which dissipates the proton gradient as heat rather than producing ATP, though this regulation is context-dependent and influenced by factors such as PGC-1α and β-adrenergic signaling. Additionally, tumors with stabilized HIF-1α often exhibit elevated local temperatures due to increased glycolytic activity and altered mitochondrial function, contributing to metabolic heat production in the tumor microenvironment. Lee et al. [49] specifically discussed HIF-1α as part of the regulatory network in BAT thermogenesis under cold-induced or hypoxic conditions, while Wang et al. [50] demonstrated how HIF-1α-driven glycolysis increases metabolic activity, indirectly contributing to heat generation in tumors.

Because Complex III is a main producer of ROS in the ETC, TSGA10 sits at a three-way nexus in the flow of electrons, protons, and ROS. Given the regulatory interaction between TSGA10 and HIF-1α [6], we hypothesize that TSGA10 is able to coordinate environmental conditions (thermal and oxygen levels) and metabolic outputs (ATP, heat, and ROS).

**Figure 4:**
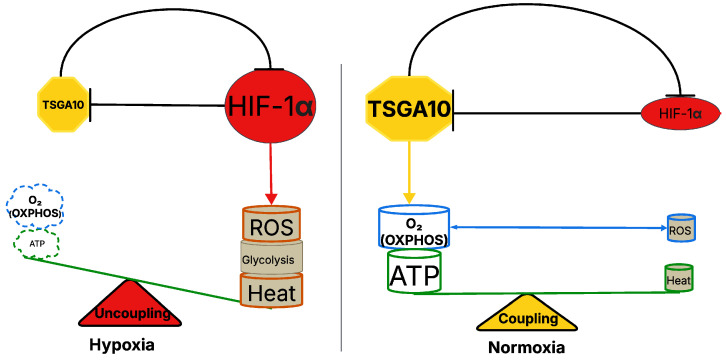
TSGA10-mediated regulation of mitochondrial coupling and metabolic balance in hypoxia and normoxia. The left panel represents the hypoxic state, where TSGA10 downregulation and increased HIF-1α activity are observed, promoting glycolysis and heat production while increasing ROS generation. This results in mitochondrial uncoupling, reducing ATP synthesis through OXPHOS. The right panel illustrates the normoxic condition, where TSGA10 enhances mitochondrial coupling, supporting efficient ATP production via OXPHOS while minimizing ROS accumulation and HIF-1α activity. The balance between mitochondrial coupling (normoxia) and uncoupling (hypoxia) is depicted as a metabolic scale, highlighting the role of TSGA10 in regulating cellular energy homeostasis.

HIF-1α is a key regulator of cellular adaptation to hypoxia, promoting glycolysis while suppressing OXPHOS [51]. The shift into glycolysis allows cells to generate ATP under low-oxygen conditions but reduces mitochondrial efficiency overall because less ATP is generated per input glucose molecule. But HIF-1α is not just activated by hypoxia: it responds to temperature also. One study on kidney tissue demonstrated that HIF-1α is significantly upregulated during cold ischemia, promoting cellular adaptations that enhance survival under low-temperature stress [52]. Another noted that HIF-1α mediates the response of skeletal muscle to cold stress [53]. These data suggest a thermal equilibrium, because TSGA10 regulates HIF-1α, suppressing its expression [2,6,7]. Furthermore, data support the counter-regulatory effect of HIF-1α acting as a suppressor of TSGA10 expression [32]. This mutual regulation makes for a tightly linked feedback loop, maintaining metabolic homeostasis and linking oxygen, ATP, and thermoregulatory roles (Figure 4).

In tightly-coupled tissues operating at cooler temperatures such as the testis, expression of TSGA10 tamps down HIF-1α, ensuring heat is not generated. However, if the temperature drops too low, HIF-1α may be activated, upregulating cellular temperature by downregulating TSGA10, thereby promoting uncoupling and heat generation. This interplay may position TSGA10 as a crucial mediator in the cellular thermoregulatory network, capable of fine-tuning mitochondrial metabolism in response to both hyperthermic and hypothermic stress. A similar logic could operate for ROS production. Under oxidative stress conditions, HIF-1α stabilization could lead to decreased expression of TSGA10, which in turn could decrease mitochondrial coupling efficiency, lowering OXPHOS and avoiding ROS toxicity. Overall, TSGA10’s inhibition of HIF-1α concretely shifts metabolic priorities (glycolysis vs. OXPHOS), enhances mitochondrial efficiency (lowered ROS/heat), and protects thermosensitive tissues (e.g., brain) by stabilizing energy and redox balance. This interaction does not merely “influence” cellular responses to hypoxia and thermal stress; it mechanistically reprograms them, dynamically adapting to the distinct needs of different tissues throughout the body. Understanding this relationship may provide insights into how cells balance energy production, oxygen availability, and thermogenesis in both normal physiology and pathological conditions like cancer.

The mutual counter-regulation between TSGA10 and HIF-1α, which integrates thermoregulation with metabolic reprogramming, has profound implications for cancer. HIF-1α, a master regulator of hypoxia adaptation, drives the Warburg effect—promoting glycolysis and suppressing OXPHOS—to fuel tumor growth in low-oxygen microenvironments. However, TSGA10’s suppression of HIF-1α introduces a critical metabolic checkpoint: by potentially stabilizing mitochondrial coupling at Complex III, TSGA10 might enhance OXPHOS efficiency, minimize ROS leakage, and reduce heat generation, thereby opposing HIF-1α’s glycolytic shift. In cancers, dysregulation of this balance could drive malignancy. For instance, loss of TSGA10 expression—common in tumors like glioblastoma [2] —may unleash HIF-1α activity, locking cells into glycolysis, exacerbating ROS production (via mitochondrial uncoupling), and fostering genomic instability. Conversely, HIF-1α’s suppression of TSGA10 under hypoxia or thermal stress (e.g., in poorly vascularized tumors) could amplify mitochondrial inefficiency, creating a pro-tumorigenic milieu of elevated ROS and heat. This ROS–heat axis may further remodel the tumor microenvironment, promoting angiogenesis or immune evasion. Notably, TSGA10’s role in thermoregulation could also explain why certain cancers thrive in thermally unstable niches (e.g., testicular tumors), where disrupted TSGA10-HIF-1α feedback loops might permit metabolic plasticity. Thus, this bidirectional repression mechanism not only underscores how tumors hijack metabolic–thermogenic crosstalk to survive stress but also highlights TSGA10 as a potential therapeutic target to restore mitochondrial fidelity and disrupt cancer’s adaptive energetics. In our model, the TSGA10 and HIF-1α feedback loop modulates cellular metabolism, favoring glycolysis, heat generation, and ROS production in hypoxia, while in normoxia the TSGA10-mediated tighter coupling of the ETC within mitochondria favors ATP synthesis, with lower heat and ROS production (Figure 4).

### 5.2. Role of Mitochondria Numbers and Blood Circulation in Thermal and Metabolic Stability

The vasculature’s role in heat distribution complements mitochondrial activity, with blood flow patterns influencing both local and global temperature regulation. For example, compared to most tissues, BAT exhibits lower numbers of mitochondria per cell, but of higher quality as each mitochondrion is highly active, leveraging UCP1-mediated uncoupling to prioritize thermogenesis over ATP synthesis, with enhanced blood perfusion facilitating heat dissipation throughout the body [54,55].

Neurons, with their high energy demands, maintain densely packed mitochondria in synapses to sustain neurotransmission, balancing quantity and quality via mitophagy, while cerebral blood flow helps maintain optimal brain temperature [45,56].

Conversely, germ cells in testes prioritize mitochondrial quality over quantity, minimizing ROS to protect genomic integrity, with specialized testicular blood flow patterns contributing to the maintenance of lower scrotal temperatures [57,58]. Pathological states, such as cancer, disrupt this balance: tumors often amplify mitochondrial numbers but exhibit dysfunctional OXPHOS, favoring glycolysis, while simultaneously altering blood vessel architecture and flow patterns [59]. Thus, tissue-specific strategies in mitochondrial quantity and activity, coupled with blood-mediated thermal transport, show adaptability to physiological and environmental stressors [60].

### 5.3. TSGA10 as a Potential Mitochondrial Regulator in Cancer

**Figure 5:**
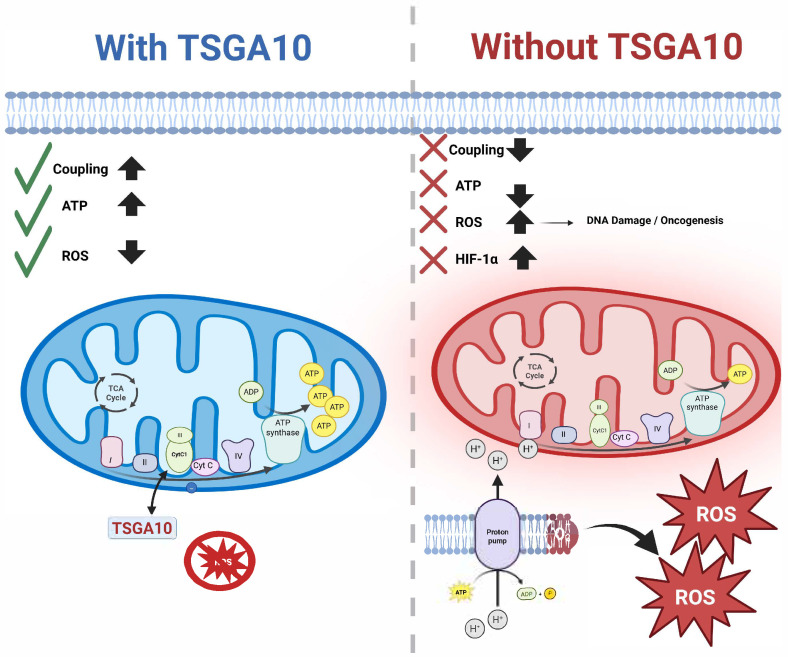
TSGA10’s interaction with CytC1 and its downstream consequences: On the **left** (blue), TSGA10 binds to CytC1 in Complex III of the mitochondrial electron transport chain, enhancing electron transfer and promoting OXPHOS. This leads to improved mitochondrial coupling, increased ATP production (↑), and reduced reactive oxygen species (ROS) levels (↓), as indicated by green checkmarks. On the **right** (red), loss of TSGA10 disrupts mitochondrial coupling (×), decreases ATP output (↓), and increases ROS production (↑), which triggers HIF-1α stabilization, DNA damage, and oncogenic signaling due to mitochondrial dysfunction and metabolic reprogramming. TSGA10.

Cancer cells exhibit metabolic plasticity, adapting their energy production depending on microenvironmental conditions such as hypoxia or nutrient stress. In some cases, tumors exploit mitochondrial uncoupling to minimize ROS accumulation, thereby preventing oxidative damage and supporting survival [61]. Given TSGA10’s hypothesized role in promoting mitochondrial coupling, its downregulation in cancer may facilitate a shift toward glycolysis, favoring the Warburg effect, where cancer cells preferentially use glycolysis over OXPHOS, even in the presence of oxygen [2]. Indeed, it has been suggested that it is the uncoupling of mitochondrial OXPHOS that drives the shift toward aerobic glycolysis of cancer cells [62] (Figure 5).

This positions TSGA10 as a potential key mediator of either oncogenesis or tumor suppression, and may help explain the fact that it has been reported to have both properties [2]. Under cold stress, cancer cells may adopt BAT-like uncoupling to reduce ROS and generate heat, sustaining proliferation [61]. Concurrently, cold impairs OXPHOS enzymes like Complex III, promoting ROS accumulation and a glycolytic shift akin to the Warburg effect [63]. This dual adaptation—uncoupling OXPHOS to survive cold and shifting to glycolysis for ATP—reflects metabolic reprogramming under thermal duress, but also describes cancer metabolism: the Warburg effect [64].

It is reasonable to speculate that aberrant thermal conditions may be a driver of oncogenic metabolic states. The duality in TSGA10 expression and metabolic activity in postmitotic versus cancer cells highlights TSGA10 as a potential metabolic “switch” that reinforces the native metabolic state of the cell—OXPHOS in postmitotic cells and glycolysis suppression in cancer. Its role in stress responses (e.g., hypoxia and drug resistance) further underscores its adaptability to the cellular context [2].

TSGA10 expression levels reflect a cell’s metabolic priorities—sustaining OXPHOS in postmitotic cells versus restraining glycolysis in cancer—making it a critical node in cellular energy homeostasis. Meanwhile, TSGA10’s dual role in mitochondrial coupling and metabolic reprogramming positions it as a pivotal regulator in cancer biology. By promoting efficient electron transfer at Complex III and suppressing ROS leakage, TSGA10 potentially reinforces OXPHOS in energy-demanding postmitotic cells. However, its downregulation in tumors—a common feature observed in cancers like glioblastoma—may facilitate mitochondrial uncoupling, a metabolic strategy that minimizes ROS toxicity while driving the Warburg effect [62,65] (Figure 5). This uncoupling not only aligns with cancer cells’ reliance on glycolysis for ATP production but also mirrors adaptations to thermal stress: under cold conditions, tumors may adopt BAT-like uncoupling to reduce ROS and generate heat, sustaining proliferation [61,63]. Here, TSGA10’s loss could act as a metabolic “key”, destabilizing OXPHOS fidelity to favor glycolysis, a hallmark of cancer metabolism. Conversely, in hypoxic or nutrient-deprived microenvironments, residual TSGA10 activity might paradoxically support tumor survival by balancing ROS and ATP production, explaining its context-dependent roles as both an oncogene and tumor suppressor. The interplay between thermal stress and TSGA10 dysfunction further underscores how aberrant environmental conditions (e.g., cold-induced OXPHOS impairment) could drive oncogenic metabolic states, akin to the Warburg effect. Ultimately, TSGA10’s ability to enforce metabolic priorities—OXPHOS in healthy cells versus glycolysis in cancer—highlights its potential as a therapeutic target to disrupt tumor metabolic plasticity and restore mitochondrial fidelity in malignancies.

### 5.4. TSGA10 Is Expressed in Postmitotic Energy-Demanding Cells

Body temperature, regulated by the hypothalamus, is maintained at approximately 37 °C, a condition under which TSGA10 exhibits minimal expression. However, mitochondria-rich postmitotic cells—such as those in the testis, retina, neurons, and nephrons—display significantly higher levels of ciliary–centrosomal proteins like TSGA10 [3,66]. These cells share common features, including a high metabolic rate, limited regenerative capacity due to their postmitotic nature, complex structure and function, temperature-sensitive ion channels, blood-barrier systems, and distinct Heat Shock Protein (HSP) expression. These metabolically active cells—especially spermatocytes—contain tightly coupled mitochondria to meet their substantial ATP demands for maintaining ion gradients, synaptic transmission (in neurons), and other energy-intensive processes. Their high coupling efficiency ensures that most energy from OXPHOS is directed toward ATP synthesis rather than heat production, minimizing proton leakage and optimizing the proton motive force for ATP generation.

In the testis, particularly in sperm cells, TSGA10 expression is exceptionally high, coinciding with a significantly lower tissue temperature (32–33 °C). While tissue temperature and TSGA10 expression are independently regulated, they operate synergistically, as a coordinated response to the high metabolic demands of sperm cells. Management of heat generation is a common theme in TSGA10-expressing cells. Excessive heat generation could lead to protein denaturation and impaired motility or function, so minimizing heat production through efficient coupling is vital for functional integrity.

In summary, some specialized postmitotic cells exhibit tightly coupled mitochondria to meet their high energy demands while minimizing thermal stress. This coupling reflects their unique functional roles and the critical need for efficient energy production, contrasting with other cell types that tolerate greater variability in energy and heat generation. The interplay between temperature, TSGA10 expression, and mitochondrial coupling underscores the sophisticated adaptations of these cells to their specific physiological environments.

## 6. Conclusions

The proposed role of TSGA10 in thermoregulation has several implications. In the testis, TSGA10’s high expression aligns with its need for precise temperature regulation during spermatogenesis. Disruptions in TSGA10 expression or function could compromise mitochondrial coupling, leading to excessive heat production and impaired fertility. In the brain, TSGA10 may protect neurons from hyperthermic damage by maintaining mitochondrial efficiency. Given the brain’s reliance on stable ion gradients and metabolic homeostasis, TSGA10 could play a crucial role in preventing heat-induced neuronal dysfunction. Meanwhile, the liver’s low TSGA10 expression may reflect its inherent tolerance to higher physiological temperatures, underscoring the tissue-specific nature of TSGA10’s thermoregulatory role. While the evidence supporting TSGA10’s role in thermoregulation and cancer is compelling, several questions remain.

Does TSGA10 directly modulate cytochrome c reductase activity? Could TSGA10 act at the intermembrane space or inner boundary membrane to influence Complex III maturation? How does HIF-1α regulation of TSGA10 vary between thermosensitive and thermally robust tissues? Investigating this interplay could reveal novel insights into tissue-specific thermoregulation. Moreover, could TSGA10 modulation be leveraged to protect against hyperthermia-induced damage in conditions such as heatstroke and febrile illnesses? In conclusion, TSGA10 represents a promising candidate in the molecular regulation of cellular thermoregulation. By promoting mitochondrial coupling and minimizing uncoupling-related heat production, TSGA10 may act as a guardian of thermosensitive tissues. The interplay between TSGA10, CytC1, and HIF-1α highlights a complex regulatory network that balances energy efficiency and thermogenesis in response to physiological demands. Future research will illuminate whether this axis can be harnessed for therapeutic interventions, offering new avenues for protecting human cells from the dual threats of hyperthermia and metabolic stress.

TSGA10’s dual association with Complex III (via CytC1) and Complex IV-linked HIF-1α pathways positions it as a critical orchestrator of mitochondrial fidelity, with profound implications for cancer. In our model, by stabilizing electron transfer at Complex III, TSGA10 can enhance mitochondrial coupling, ensuring efficient ATP synthesis while minimizing ROS leakage—a safeguard against genomic instability. As previously reported [2], TSGA10 expression exhibits two distinct phases—downregulation and upregulation—during cancer progression. TSGA10 overexpression may indicate a compensatory action of TSGA10 and its tumor suppressor functionality. However, in a phase of cancer progression where TSGA10 is downregulated (e.g., glioblastoma), perhaps under HIF-1α pressure, disrupted coupling at Complex III may promote electron leakage, elevating ROS to mutagenic levels that drive oncogenic mutations. Concurrently, TSGA10’s mutual counter-regulation with HIF-1α hypoxia-activated promotion of glycolysis—creates a metabolic tug-of-war. In tumors, HIF-1α dominance suppresses TSGA10, locking cells into the Warburg effect [62], while TSGA10 deficiency might destabilize Complex III, exacerbating mitochondrial dysfunction and ROS-driven stress adaptation. This bidirectional axis may explain TSGA10’s paradoxical roles in cancer: in thermosensitive tissues like the testis, its high expression could suppress HIF-1α to maintain OXPHOS and thermal stability, protecting against malignant transformation, whereas in thermally resilient tissues like the liver, low TSGA10 levels may permit HIF-1α-driven glycolytic reprogramming, facilitating tumorigenesis. The tissue specificity of TSGA10’s function—critical in brain and testes but dispensable in liver—mirrors cancer vulnerabilities, where loss of TSGA10 in thermoregulatory tissues could permit heat-induced microenvironmental remodeling (e.g., angiogenesis and immune evasion). Thus, TSGA10 emerges as a metabolic–thermogenic rheostat, whose dysregulation at Complex III and crosstalk with HIF-1α may underpin the metabolic plasticity that fuels carcinogenesis, offering novel strategies to target mitochondrial vulnerabilities in tumors.

## Data Availability

Data generated and analyzed during this study, including all raw and processed data from the transfection experiments, are available upon reasonable request from the corresponding author.

## Abbreviations

The following abbreviations are used in this manuscript:

TSGA10	Testis-Specific Gene 10
HIF-1α	Hypoxia-Inducible Factor 1-Alpha
VEGFA	Vascular Endothelial Growth Factor A
MMP2	Matrix Metalloproteinase-2
MMP9	Matrix Metalloproteinase-9
OXPHOS	Oxidative Phosphorylation
ROS	Reactive Oxygen Species
ETC	Electron Transport Chain
CytC1	Cytochrome c1
UCP1	Uncoupling Protein 1
BAT	Brown Adipose Tissue
MICOS	Mitochondrial Contact Site and Cristae Organizing System
ATP	Adenosine Triphosphate
HSPs	Heat Shock Proteins
RISP	Rieske Iron–Sulfur Protein
COX4	Cytochrome c Oxidase Subunit 4
